# Cervical Cancer: 90-70-90 and Palliative Care

**DOI:** 10.1200/GO.21.00230

**Published:** 2021-09-30

**Authors:** James F. Cleary

**Affiliations:** ^1^ Walther Global Palliative Care and Supportive Oncology, Indiana University Simon Comprehensive Cancer Center, Indianapolis, IN

In August 2020, in the midst of the COVID-19 pandemic, the WHO outlined a global strategy to accelerate the elimination of cervical cancer, a public health problem.

- 90% of girls fully vaccinated with human papillomavirus (HPV) vaccine by age 15 years.
- 70% of women are screened by 35 years or again and again by 45 years.
- 90% of women identified with cervical disease receive treatment.

Both the number of women developing cervical cancer and those dying from the disease will be reduced with a 67% reduction in incidence together with 62 million cervical cancer deaths averted by 2120. Over ambitious? Not really. With the implementation of HPV vaccination in girls in 2007 and young boys in 2013 throughout Australia, it is estimated that cervical cancer will be eliminated by 2035. Very impressive outcomes surely make HPV vaccines an anticancer vaccine.

Until there is a successful implementation of vaccination and screening, cervical cancer will continue to have a continued presence worldwide, particularly in low- and middle-income countries (LMICs) where there will be both higher incidence rates and death rates. Women living with HIV infection are at increased risk for chronic HPV infection and are six times as likely to develop HPV-associated cervical cancer compared with HIV-negative women. The WHO panel has set very bold targets to be met by 2030 with 90% of women with identified cancer managed. Careful wording, as 85% of these women live in LMICs, many of whom may never be identified as having cervical cancer. The panel calculated in 2017 that over 250,000 died from cervical cancer (decedents) and 2.5 million women who will have the disease but will not die in a given year (nondecedents) needed palliative care. Although not on the program's overview webpage,^1^ the WHO in this series of papers has addressed the palliative care needs of these patients, the reduction of suffering for these women and their families.

Krakauer et al,^2-4^ using a mixture of tools, including a modified Delphi process, calculate the level of suffering. One could question the methodologies used: Was the focus group large enough? Were the right people surveyed? Could patients have been included in the Delphi process? Regardless, it is not unreasonable to accept the overall burden of symptoms they have established. Eighty-five percent of decedents had moderate to severe pain, whereas < 5% had no pain. Significant vaginal discharge and bleeding were found to occur in approximately 2/3 of decedents. Pain was also a significant issue in nondecedents while anxiety and depression were common in both groups. Sexual dysfunction was common across the disease trajectory (> 80%) with over 40% abandoned by their intimate partner. Financial distress was a significant consideration in decedents, nondecedents, and family caregivers. Stigmatization is a significant issue, half of the decedents experience loss of meaning of life and a third suffered loss of faith because of their disease. The authors have probably understated the impact on caregivers by assuming only one person affected for each patient. For many, the number affected could be much greater, with parents and children being impacted.

Therefore, cervical cancer causes a great deal of suffering in those living with and dying of the disease. Interestingly, it is only in the second paper that the authors point out that their estimates of suffering are much greater than those found by the Lancet Commission for Palliative Care and Pain Relief.^5^ Cervical cancer is a great example of cancer control in its entirety as we can prevent the disease, detect it early, and treat the disease and we can also provide significant palliation. Palliative care for cervical cancer will remain a significant issue worldwide.

The second^3^ and third^4^ papers follow with both essential and an augmented packages of palliative components that should be provided. Importantly, the essential package is not a poor person's palliative care for those who cannot afford it. It is a starting point. One can question the inclusions of some items which many may not consider part of health care, for example, a flashlight or the use of adult diapers. Some of these become critical parts of health care when they contribute greatly to physical, psychological, and social suffering. Fistulas (vesiculovaginal, rectovaginal, or both) resulting from cancer can be a major cause of this suffering and not easily repaired even in well-resourced countries. A diaper may be indispensable for many women.

The authors address the treatment of pain and the need to have opioids, controlled medicines, sadly lacking in many LMICs, despite continued efforts to ensure access in LMICs. They also address the issue of neuropathic pain and the need for adjuvants. They comment that oral amitriptyline and dexamethasone can be used to improve analgesia (dexamethasone is not just for neuropathic pain; it is particularly useful to reduce edema that may be contributing to pain). The studies that support amitriptyline as a neuropathic agent show a *number needed to treat* of 5:1; they may only be effective in 20% of people. This raises the question as to whether this is something that should be an essential medicine, but if they are cheaply available for other purposes such as the treatment of depression, they may be useful. The authors make mention of medicines such as haloperidol, which can be used for many purposes including nausea and vomiting as well as delirium. Personal reports of this not being available in sub-Saharan Africa raises the issue as to the future availability of these products if pharmaceutical companies do not make them available.

The issue of spirituality within the health care system is significant. Although the authors stress the ability of any clinician to be able to screen for spiritual distress, the engagement of local spiritual care providers becomes very important. However, the education of spiritual providers with an understanding of both cancer care and palliative care is important. The authors rightly point out that stigma and even guilt or shame may come in relationships with a spiritual counselor. In fact, there may be times when this is actually directed from the spiritual counselors themselves.

The WHO team then goes on to propose an augmented package that offers modes of care beyond the essential package with evidence provided for many therapies.^4^ Surgery is commonly available throughout the world, but oncological surgery is often complex and requires great expertise. Correctly, it is pointed out that a colostomy may circumvent issues of a rectal-vaginal fistula rather than attempting surgery within the tumor bed.

There may be an advantage with the spinal administration of opioids. The randomized study by Smith et al^6^ identified that patients with pelvic malignancy may be particularly responsive to the intraspinal administration of analgesics. While that study was industry-sponsored, with implantable pumps, an intrathecal catheter can be placed, tunneled subcutaneously and externalized, and used for the administration of analgesics at a much lower cost but still with the required expertise. Increased levels of expertise are required when using medications such as extended-release morphine, fentanyl patches, and methadone, and ketamine and lidocaine. Being able to consult with palliative care specialists with that experience and training is imperative.

A major question needs to be asked as to the place of radiation therapy. Radiation therapy is not readily available, but the authors recognize that it often provides rapid relief of the frequent symptoms of pain, vaginal bleeding, and discharge (> 60% of decedents). Many people in the world are lacking access to radiation therapy as with other modes of cancer care and control, especially in LMICs.^7^ Perhaps the recognition of radiation therapy's essential role in the management of both breast and cervical cancers will aid in making this more available.

The authors also make significant advances in the field of palliative care implementation. They define days with a palliative care encounter as a more accurate and useful measure than days in palliative care, as described by Knaul et al.^5^ This is critical in terms of establishing the needs for palliative care services and their appropriate resourcing, but if services are limited and a patient is suffering, then perhaps we are underestimating the days of the suffering of the lived experience.

Palliative care should be a part of universal health care and is, in fact, one of the least costly components. It should therefore be included in universal health coverage such that a patient should receive these without suffering financial hardship. Does this cart before the horse? Does one need palliative care to be provided before one has universal health coverage or does, in fact, the need for palliative care become a driver for universal health coverage?

This series of papers is an essential addition to the palliative care and global oncology literature. Although these may not answer the authors' hypothesis that moderate and severe suffering is more prevalent and multifaceted among women with cervical cancer than among people with other cancers or serious illnesses, they certainly make a strong case for the widespread nature of suffering related to cancer of the cervix. In terming the current situation morally indefensible neglect, they make a strong case for the improved prevention and greater relief of suffering related to the disease. This is not just needed in LMICs but in all parts of the globe where cervical cancer has not yet been controlled.
